# A Rare Case of Large Cell Neuroendocrine Carcinoma of the Thymus Presenting as a Lower Neck Mass in a Young Male Patient

**DOI:** 10.7759/cureus.115920

**Published:** 2026-09-07

**Authors:** Kaushik Saha, Aparna Thakur, Debayan Dasgupta

**Affiliations:** 1 Department of Pathology, Murshidabad Medical College and Hospital, Berhampore, IND; 2 Department of Radiology, M L Jain Diagnocare, Berhampore, IND

**Keywords:** ihc markers, large-cell neuroendocrine carcinoma, lcnec, thymic lcnec, thymus

## Abstract

Large cell neuroendocrine carcinoma (LCNEC) of the thymus is a strikingly unusual and high-grade thymic tumor with a poor prognosis. Studies with large series of patients on the clinical, epidemiological, and biological behavior of the disease are not available due to its low prevalence and short survival. We report here such a rare case of LCNEC in a 33-year-old male patient who primarily presented with a mass in the suprasternal and left supraclavicular regions with multiple bilateral cervical lymph nodes. Fine needle aspiration cytology from the neck mass demonstrated hypercellular smears with numerous variably sized, loosely cohesive clusters, including large hypercellular ones, of medium-sized atypical cells with scant cytoplasm, hyperchromatic nuclei, and granular chromatin, with a few having small, inconspicuous nucleoli. Peripheral nuclear palisading or rosette-like structures were noted in many places. An anterior mediastinal invasive mass lesion, possibly of thymic origin, extending to the neck and encasing the major vessels, including branches of the aortic arch, without any evidence of lung parenchymal extension, with multiple bilateral cervical lymph nodes was identified on contrast-enhanced computed tomography. Incisional biopsy revealed large atypical cells mostly arranged in nests, solid sheets, and trabeculae, with hyperchromatic nuclei, coarse chromatin, a moderate amount of eosinophilic cytoplasm, frequent mitoses, and apoptotic bodies. The cells were immunopositive for pancytokeratin, EMA, synaptophysin, chromogranin A, CD56, and TTF-1, with a high Ki-67 labeling index, while negative for all other relevant markers. Misdiagnosis or delay in the correct diagnosis of this rare but aggressive thymic tumor may lead to disease spread and progression to systemic involvement. Many such cases of thymic LCNEC need to be studied to design and optimize standard management.

## Introduction

Thymic neuroendocrine neoplasms (TNENs) account for 2%-5% of all thymic neoplasms and only 0.4% of all neuroendocrine neoplasms (NENs), having many similarities with pulmonary NENs [1]. Large cell neuroendocrine carcinoma (LCNEC) of the thymus is a strikingly unusual high-grade tumor, constituting 14%-26% of all TNENs [2]. Studies with large series of patients on the clinical, epidemiological, and biological behavior of the disease are not available due to its low prevalence and short survival [3,4]. We report here such a rare case of LCNEC in a 33-year-old male patient.

## Case presentation

A 33-year-old male patient primarily presented with a mass in the suprasternal and left supraclavicular regions with multiple bilateral cervical lymph nodes. Fine needle aspiration cytology (FNAC) from the neck mass demonstrated (Figure 1) hypercellular smears with numerous variably sized, loosely cohesive clusters, including large hypercellular ones, of medium-sized atypical cells with scarce cytoplasm, hyperchromatic nuclei, and granular chromatin, with a few having small, inconspicuous nucleoli. Peripheral nuclear palisading or rosette-like structures were noted in many places. Necrosis and naked nuclei were observed in the background.

**Figure 1:**
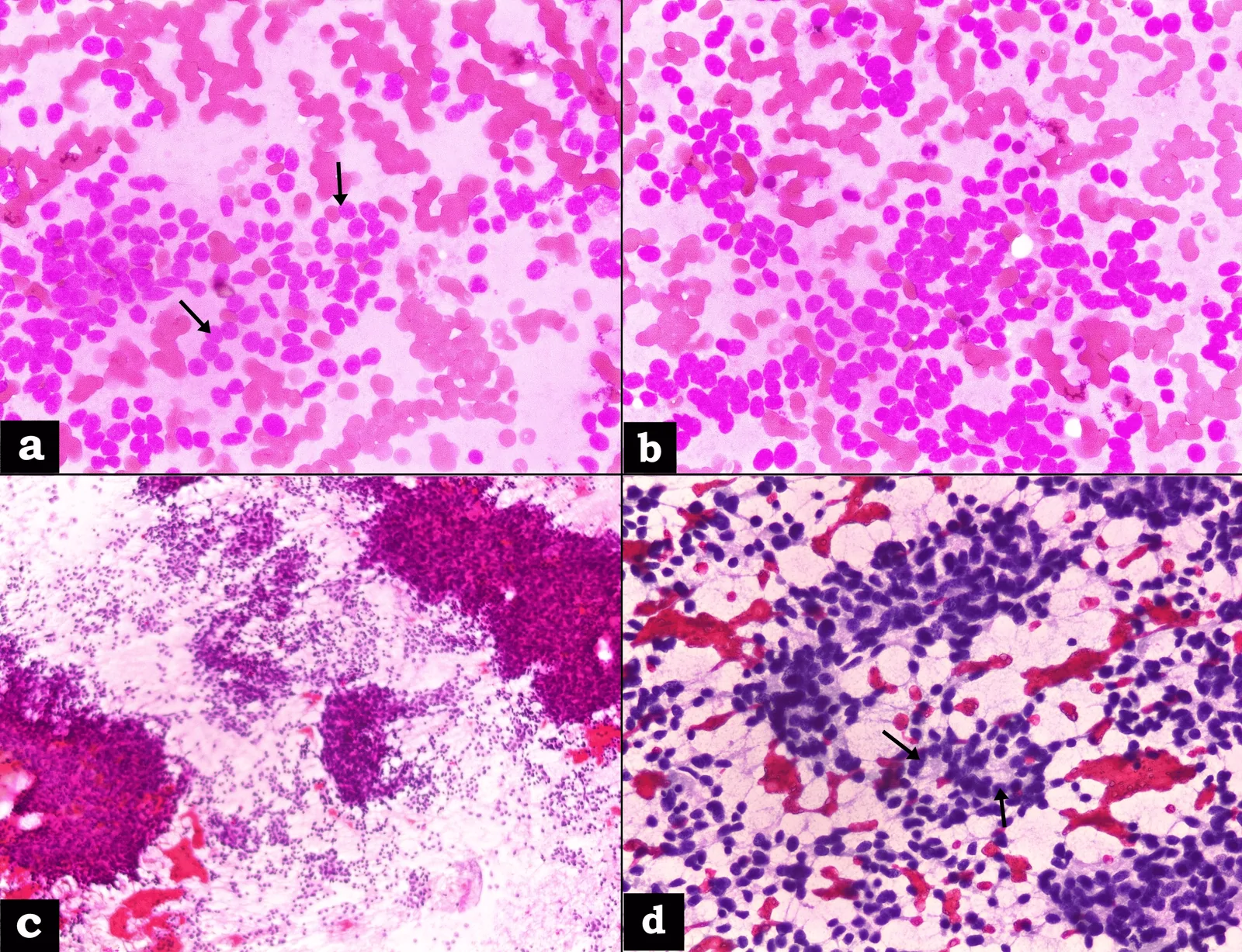
Photomicrographs of cytology smears (a) (MGG, ×400), (b) (MGG, ×400), (c) (Pap, ×100), and (d) (Pap, ×400) showing variably sized, loosely cohesive clusters of cells with scarce cytoplasm and hyperchromatic nuclei, forming rosette-like structures (marked by arrows) in places MGG: May-Grünwald Giemsa; Pap: Papanicolaou

Contrast-enhanced computed tomography (CECT) (Figure 2) identified an anterior mediastinal invasive mass lesion, possibly of thymic origin, extending to the neck and encasing the major vessels, including branches of the aortic arch, without any evidence of lung parenchymal extension, along with multiple bilateral cervical lymph nodes.

**Figure 2:**
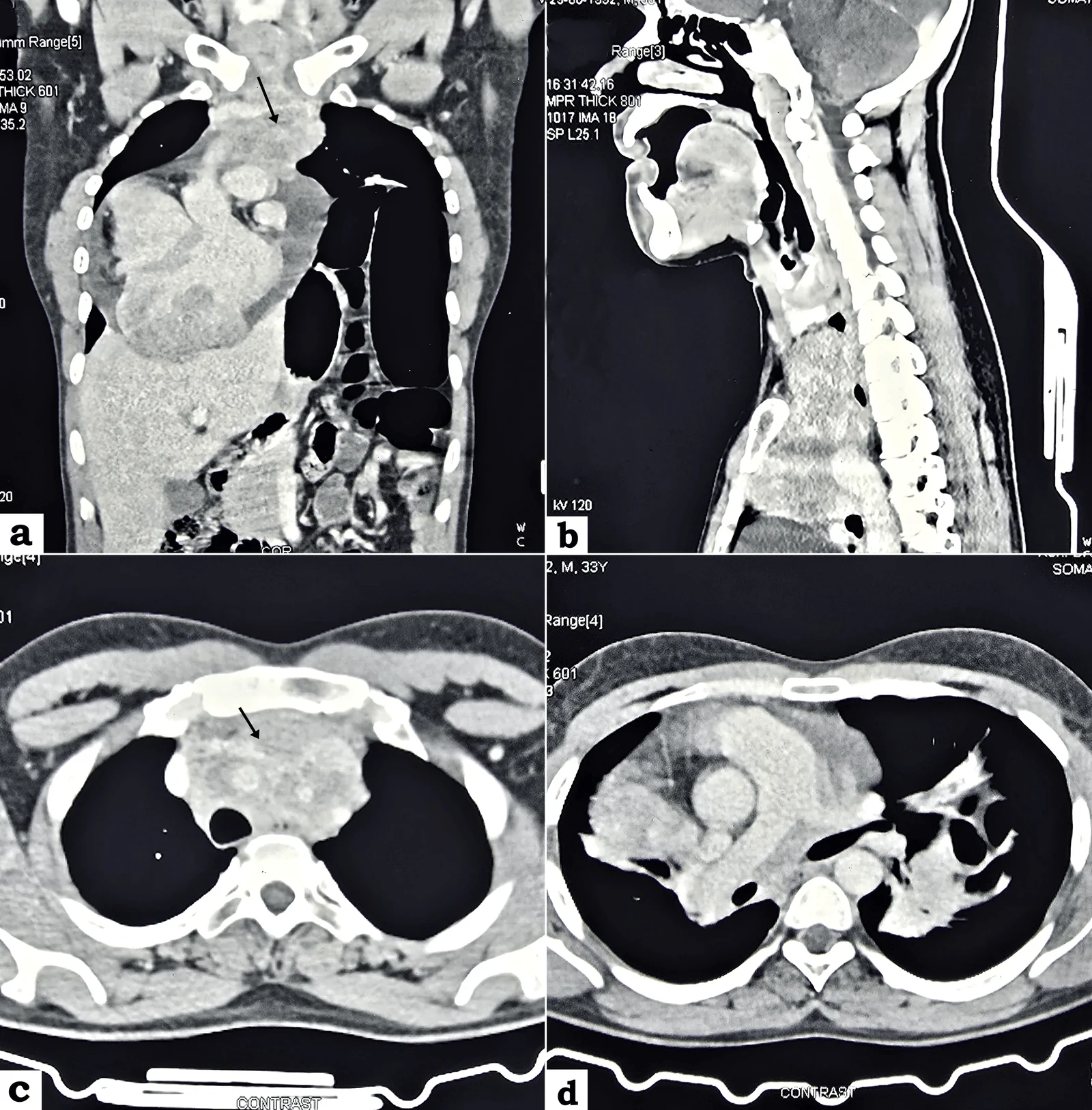
(a) Coronal, (b) sagittal, and (c), (d) axial views of the CT scan of the chest showing a huge anterior mediastinal mass (marked with arrows) extending to the neck and encasing the major vessels CT: computed tomography

Incisional biopsy (Figure 3) revealed large atypical cells mostly arranged in nests, solid sheets, and trabeculae, with hyperchromatic nuclei, coarse chromatin, a moderate amount of eosinophilic cytoplasm, frequent mitoses, and apoptotic bodies. Foci of necrosis and peripheral palisading were also noted.

**Figure 3:**
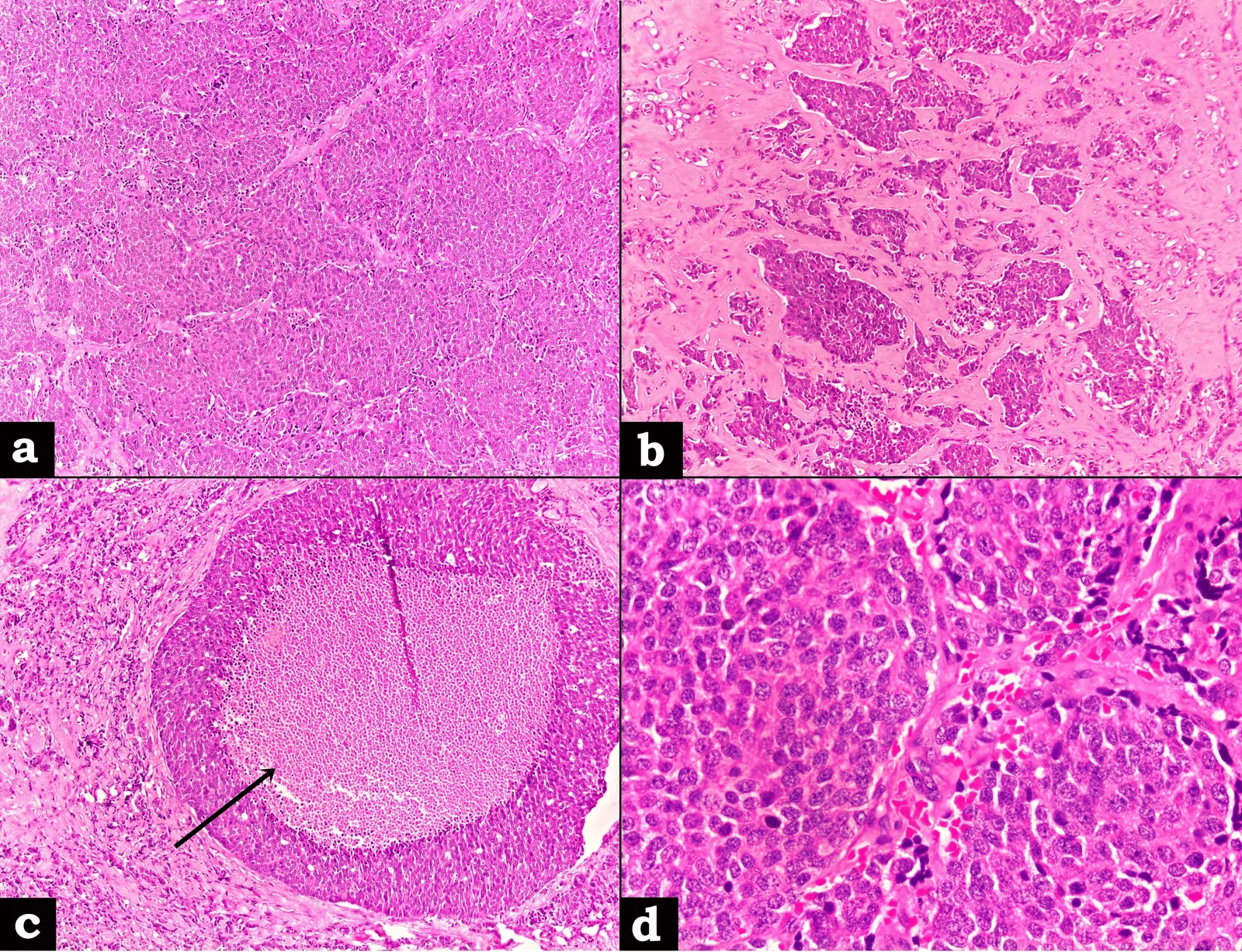
Photomicrographs showing low-power views (a), (b), and (c) (H&E, ×100) and high-power view (d) (H&E, ×400) of atypical cells arranged in nests, solid sheets, and trabeculae, with hyperchromatic nuclei, frequent mitoses, apoptotic bodies, and foci of necrosis (marked with an arrow) H&E: hematoxylin and eosin

The cells were immunopositive (Figure 4) for pancytokeratin, EMA, synaptophysin, chromogranin A, CD56, and TTF-1, with a high Ki-67 labeling index (35%), while negative for all other relevant markers, such as CD117, CD5, p63, p40, CK7, and Napsin A.

**Figure 4:**
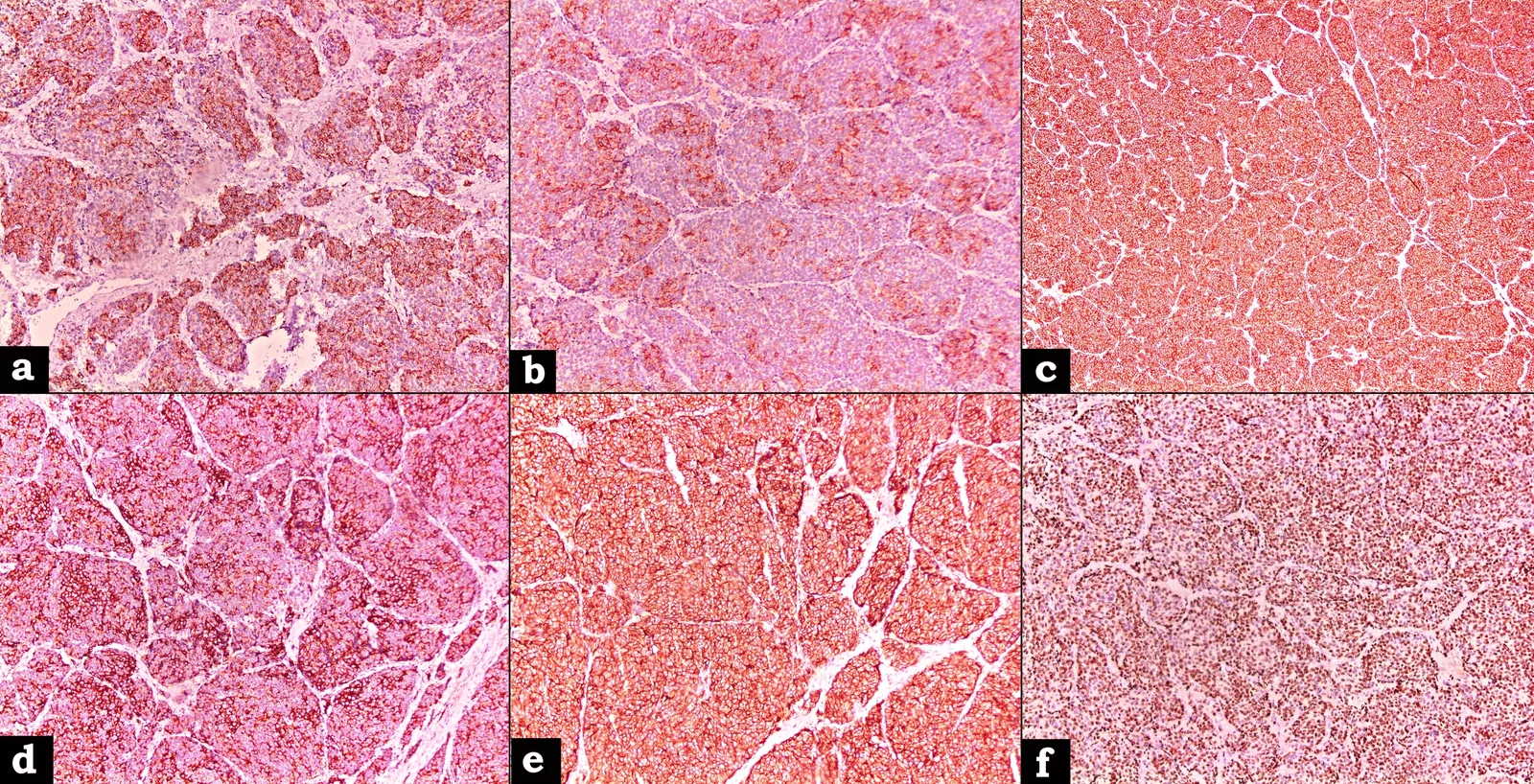
Positive immunostaining of neoplastic cells with (a) pancytokeratin (×100), (b) EMA (×100), (c) synaptophysin (×40), (d) chromogranin A (×100), (e) CD56 (×100), and (f) TTF-1 (×100)

Positron emission tomography with 18F-fluorodeoxyglucose (FDG PET/CT) showed high FDG uptake in the lesion.

## Discussion

LCNEC is a high-grade thymic tumor comprising large cells rather than non-small cells, with neuroendocrine morphology, a high mitotic rate, almost universal presence of necrosis, and positivity for neuroendocrine immunohistochemical (IHC) markers. It is more common in adult males. Around 50% of patients are asymptomatic at the time of initial presentation, as in our case [5,6]. TNENs are morphologically indistinguishable from pulmonary NENs in each category. Differentiation from pulmonary NENs depends almost entirely on clinical information and radiological studies. Thymic carcinoma also needs to be differentiated from LCNEC, preferably by IHC markers, as thymic carcinoma usually does not demonstrate any neuroendocrine architecture. Thymic carcinomas are commonly positive for CD117, CD5, and p63, in contrast to LCNEC [3,7]. Ahn et al. [8] reviewed 21 patients with surgically resected primary thymic neuroendocrine tumors and found only three cases of LCNEC, all of which showed lymphatic emboli. Yoon et al. [9] reported two cases of surgically resected LCNEC of the thymus that subsequently developed metastasis and recurrence. Fewer than 50 cases of LCNEC of the thymus have been reported in the literature to date, and most of them were diagnosed on resected specimens, except for a few diagnosed by incisional or tru-cut biopsy [2,4,10]. Multimodality treatment consisting of complete surgical resection with postoperative adjuvant chemoradiation therapy is recommended depending on the patient profile [10,11].

## Conclusions

As the clinical and pathological data are scarce, the chances of misdiagnosis or delay in the correct diagnosis may lead to local invasion and systemic involvement. Hence, prompt diagnosis and treatment are essential with a multidisciplinary approach. Many such cases of thymic LCNEC need to be documented and studied to design and optimize the diagnostic work-up and management.
